# Social Support and Quality of Life in Hemodialysis Patients: A Comparative Study with Healthy Controls

**DOI:** 10.3390/medicina60111732

**Published:** 2024-10-22

**Authors:** Leszek Sułkowski, Andrzej Matyja, Maciej Matyja

**Affiliations:** 1 Department of General Surgery, Regional Specialist Hospital, 42-218 Częstochowa, Poland; leszeksulkowski@szpitalparkitka.com.pl; 2 2nd Department of General Surgery, Jagiellonian University Medical College, 30-688 Kraków, Poland; andrzej.matyja@hipokrates.org

**Keywords:** social support, quality of life, hemodialysis, end-stage renal disease, chronic kidney disease, Modified Social Support Survey (MSSS), Modified Social Support Survey—5-item version (MSSS-5), World Health Organization Quality of Life questionnaire (WHOQOL-BREF)

## Abstract

*Background and Objectives*: Hemodialysis patients face significant physical and psychological challenges, including diminished quality of life and reduced social support. This study aimed to assess the levels of social support and quality of life in hemodialysis patients and identify the sociodemographic and dialysis-related factors influencing these outcomes. *Materials and Methods*: This study included 115 hemodialysis patients and 107 healthy controls. Social support was measured using the Modified Social Support Survey (MSSS) and its abbreviated version (MSSS-5). Quality of life was assessed using the WHOQOL-BREF questionnaire. Demographic variables (age, sex, education, marital status) and dialysis-related factors (session duration, Kt/V, vascular access type, and urea reduction ratio) were analyzed to determine their effects on social support and quality of life. *Results*: Hemodialysis patients reported significantly lower scores in the Physical Health and Psychological Health domains of the WHOQOL-BREF compared to healthy controls. Males on hemodialysis scored lower than the controls in the Physical Health, Psychological Health, and Environment domains of the WHOQOL-BREF and the Affectionate Support and Positive Social Interaction subscales of MSSS. Conversely, hemodialysis females reported higher scores for Tangible Support, Emotional/Informational Support, and Affectionate Support. Longer dialysis sessions negatively impacted the Social Relationships domain. Married hemodialysis patients had higher Emotional/Informational Support and Affectionate Support scores. *Conclusions*: Hemodialysis patients experience diminished physical and psychological quality of life, particularly males. Social support, especially emotional and informational support, is crucial for hemodialysis patients, with marital status playing a key role. Addressing these psychosocial factors may improve outcomes for hemodialysis patients.

## 1. Introduction

End-stage renal disease is a severe, chronic condition characterized by the irreversible loss of kidney function, requiring life-sustaining therapies such as hemodialysis or kidney transplantation. While kidney transplantation is considered the gold standard for improving quality of life and life expectancy, hemodialysis remains the most common treatment modality worldwide, with approximately 89% of dialysis patients receiving this form of therapy [1,2]. Despite significant advancements in dialysis technology and care, patients on hemodialysis continue to face numerous challenges, including high morbidity and mortality rates, frequent hospitalizations, and a diminished health-related quality of life [2,3]. These issues are exacerbated by the physical, emotional, and social burdens of living with a chronic disease that often restricts patients’ ability to lead normal lives.

Patients undergoing hemodialysis must adhere to rigorous treatment regimens, including dietary restrictions, fluid limitations, and frequent dialysis sessions, all of which require major lifestyle adjustments [4]. The burden of these demands, coupled with the debilitating symptoms of end-stage renal disease—such as fatigue, insomnia, pain, and depression—profoundly impacts patients’ physical and mental well-being [2,5,6]. Physical limitations often manifest as impaired mobility and a reduced ability to perform daily activities, while mental health challenges frequently include depression, anxiety, social isolation, and a lack of emotional well-being [3,6].

In this context, social support emerges as a crucial factor in mitigating the negative effects of end-stage renal disease and improving patient outcomes. Social support refers to the emotional, informational, and practical assistance provided by family, friends, healthcare providers, and broader social networks [7,8]. Strong social support systems have been associated with better outcomes in chronic disease management, including improved adherence to treatment, enhanced psychological well-being, and reduced disease burden [7,9,10]. Specifically, in hemodialysis patients, higher perceived social support has been linked to improved health-related quality of life, reduced depressive symptoms, and better coping with the emotional and physical demands of the disease [11,12]. Social support not only aids patients in managing daily treatment challenges but also reduces feelings of social isolation and fosters psychological resilience [8,13].

Social support is typically categorized into emotional, instrumental, informational, and appraisal support [7,12]. Emotional support involves expressions of empathy and understanding, while instrumental support refers to practical help with daily tasks. Informational support includes guidance and advice, and appraisal support involves feedback, helping patients evaluate their situation. Among these, emotional and instrumental support is particularly critical for hemodialysis patients, who often rely on family members or caregivers for both practical assistance and emotional comfort in coping with their illness [12,14]. Research shows that patients with higher levels of perceived social support experience greater satisfaction with their care, fewer hospitalizations, and better adherence to treatment protocols [7].

Psychological disorders, particularly depression and anxiety, are highly prevalent among hemodialysis patients and have a detrimental impact on both physical and mental health outcomes [6,12]. Depression affects an estimated 20–30% of end-stage renal disease patients, leading to poorer health-related quality of life, increased hospitalization risk, and higher mortality rates [12]. Social support has been shown to alleviate depressive symptoms in this population by providing emotional comfort and fostering coping strategies to manage mental health challenges [8,9,14]. Studies demonstrate that patients who perceive high levels of social support are more resilient, less prone to severe depression, and are more likely to maintain a positive outlook on their condition [8,14]. Additionally, religious and spiritual involvement often enhances patients’ perceptions of social support, further improving their psychological well-being [15].

In addition to its psychological benefits, social support has been linked to better physical outcomes in hemodialysis patients. Those with strong social support are more likely to adhere to treatment regimens, including dietary restrictions and medication, which are essential for managing end-stage renal disease [4,7]. This adherence leads to improved physical health outcomes, such as reduced fatigue and fewer complications [9]. Fatigue, one of the most debilitating symptoms of hemodialysis, significantly affects both physical and mental quality of life and is often a predictor of poorer treatment outcomes and decreased survival [9]. The relationship between social support and reduced fatigue highlights the importance of addressing this issue in patient care.

Despite the well-documented benefits of social support, many hemodialysis patients experience a lack of it, particularly those who are socially isolated or without strong family or community networks [11,16]. Social isolation is common, as many hemodialysis patients are unemployed due to their illness and have limited social interactions, exacerbating feelings of depression and anxiety and further diminishing their quality of life [2,11]. Identifying patients at risk of inadequate social support is essential for developing interventions that can improve their well-being [11].

In recent years, there has been a growing recognition of the need for patient-centered care approaches that address the psychosocial as well as medical needs of end-stage renal disease patients [2,7,17]. Social support interventions, including psychoeducational programs, support groups, and caregiver support, have proven effective in improving both mental and physical health outcomes in hemodialysis patients [7]. Healthcare providers play a crucial role in fostering social support by creating environments that encourage patient engagement and by facilitating connections to support networks [7,13,16]. These efforts are particularly important given the rising global population of dialysis patients and the increasing costs associated with their care, necessitating more efficient and comprehensive care models that address the full spectrum of patient needs [2].

Given the significant impact of social support on the well-being of hemodialysis patients, it is essential to identify factors that influence the level of social support in this population. Understanding these factors can help healthcare providers develop targeted interventions to improve social environments and enhance the quality of life for hemodialysis patients. This study aims to evaluate the level of social support among hemodialysis patients and to explore the sociodemographic and health-related factors that affect it. By addressing the gap in research on the social support needs of this population, this study aims to contribute to the development of more comprehensive care strategies that prioritize psychosocial well-being alongside clinical care. Additionally, this study explores the potential utility of a shortened questionnaire for assessing social support in clinical practice.

## 2. Materials and Methods

Participants were recruited from the Dialysis Unit of the Regional Specialist Hospital in Częstochowa, Poland. All participants were informed about the study, and the inclusion criteria were as follows: diagnosis of end-stage renal disease in patients undergoing hemodialysis, willingness to participate, and provision of informed consent. Exclusion criteria included being under 18 years of age, having acute kidney failure, refusing to participate, or incomplete questionnaire responses. The study was approved by the Regional Bioethical Committee (K.B.Cz.-0014/2017).

A total of 115 hemodialysis patients met the inclusion criteria. These patients underwent hemodialysis three times a week and had a median age of 63.7 years (SD = 11.5); 79 were male (68.7%). The control group consisted of 107 healthy individuals who were free from kidney disease and free from chronic conditions requiring more specialized treatment than care by a general practitioner, including staff members of the Dialysis Unit and their relatives, with a median age of 56.2 years (SD = 10.8), and 72 were males (67.3%) (Table 1).

To evaluate social support in hemodialysis patients, the Modified Social Support Survey (MSSS) and its abbreviated 5-item version (MSSS-5) were administered, along with the World Health Organization Quality of Life questionnaire (WHOQOL-BREF). The MSSS and MSSS-5 questionnaires were used with permission from the National Multiple Sclerosis Society, and the WHOQOL-BREF was used with formal approval from the World Health Organization (WHO). Both hemodialysis patients and healthy controls completed these questionnaires.

The MSSS is a multidimensional tool that measures perceived social support based on the Medical Outcomes Study. It assesses perceived social support in four dimensions: Tangible Support, Emotional/Informational Support, Affectionate Support, and Positive Social Interactions [4]. The full 18-item version of the MSSS and its 5-item abbreviated version were used in this study.

The WHOQOL-BREF evaluates self-perceived quality of life across four domains: Physical Health, Psychological Health, Social Relationships, and Environment, as well as two questions on the overall quality of life and health [18,19,20,21]. These domains cover a wide range of areas, including daily activities, energy, pain, self-esteem, social support, and environmental factors such as access to healthcare and financial resources [19,20,22]. The demographic variables (e.g., sex, age, education, marital status), comorbidities (diabetes and hypertension), and dialysis-related variables (e.g., session duration, months on dialysis, Kt/V, vascular access, and urea reduction ratio [URR]) were analyzed to explore their effects on MSSS, MSSS-5, and WHOQOL-BREF scores (Table 2, Table 3 and Table 4).

### Statistical Analysis

Frequency, range, and means were calculated for demographic data, with standard deviations (SDs) provided for continuous variables. Comparisons of continuous variables were performed using Student’s *t*-test, while comparisons across three or more groups were conducted using ANOVA. A *p*-value of ≤0.05 was considered statistically significant. Cronbach’s alpha was used to reflect the internal consistency of the MSSS items within our sample. A Cronbach’s alpha value above 0.7 is considered acceptable, and values above 0.8 indicate good reliability. For the correlation analysis between the MSSS-5 and the full MSSS, Pearson’s correlation coefficient was used.

## 3. Results

A total of 115 hemodialysis patients (aged 30 to 86 years) from a single dialysis unit, were compared to 107 healthy controls. Hemodialysis patients scored significantly lower than controls in the Physical Health and Psychological Health domains of the WHOQOL-BREF (*p* < 0.0001 for both). No significant differences were observed between the two groups in the remaining WHOQOL-BREF subscales, MSSS, or MSSS-5 questionnaires (Table 1).

The influence of sex, age, education level, and marital status on social support (MSSS) and quality of life (WHOQOL-BREF) was analyzed (Table 2). Male hemodialysis patients scored lower than male controls in the Physical Health, Psychological Health, and Environment domains of the WHOQOL-BREF, and in the Affectionate Support and Positive Social Interaction subscales of the MSSS, including both the full and abbreviated versions. In contrast, female hemodialysis patients scored higher than healthy females in the Tangible Support, Emotional/Informational Support, and Affectionate Support subscales of the MSSS and MSSS-5.

To assess the impact of age, patients were divided into two groups using a median age of 65 years. No significant differences were found between younger and older patients for the MSSS or WHOQOL-BREF scores (Table 2).

When education level was examined, statistically significant differences were found in the Physical Health and Environment domains of the WHOQOL-BREF. However, no significant effects of education were found in MSSS scores (Table 2).

Marital status had no significant impact on WHOQOL-BREF scores. However, married hemodialysis patients had significantly higher scores in the Emotional/Informational Support, Affectionate Support, and Positive Social Interaction subscales of MSSS, as well as higher total MSSS and MSSS-5 scores (Table 2).

Among the two most commonly reported comorbidities, diabetes and hypertension, we found statistically significantly worse results in the Social Relationships and Environment domains of the WHOQOL-BREF test in the group of patients with diabetes. Additionally, we observed significantly worse scores for the MSSS and MSSS-5 tests, as well as in the Emotional/Informational and Positive Social Interactions subscales, for the group of patients without hypertension (Table 3).

Dialysis-related factors, such as the duration of a single hemodialysis session, months on hemodialysis, Kt/V, type of vascular access, and URR, were also analyzed (Table 4). Patients with dialysis sessions lasting more than 4 h (*n* = 22) had lower scores in the WHOQOL-BREF Social Relationships domain, though no significant differences were found in MSSS scores. We also did not find a statistically significant correlation between the number of months of dialysis therapy and the MSSS test, its subscales, or the domains of the WHOQOL-BREF test.

Patients with central venous catheters for dialysis had higher scores on the Tangible Support subscale of MSSS compared to those with arteriovenous fistulas. However, no significant differences were observed between these groups in other MSSS subscales or WHOQOL-BREF domains (Table 4).

When comparing patients based on URR values (above or below the median), no statistically significant differences were found in any of the questionnaire scores, including MSSS, MSSS-5, and WHOQOL-BREF (Table 4).

The calculated Cronbach’s alpha for the MSSS test was 0.88, which indicates a high level of internal consistency. The Pearson correlation coefficient between the MSSS-5 and full MSSS scores was calculated to be 0.80. This indicates a strong positive correlation between the two tests, suggesting that the MSSS-5 test provides a reliable estimate of full MSSS scores.

## 4. Discussion

Chronic kidney disease is a growing global public health issue, with rising rates of end-stage renal disease and increased life expectancy among affected populations [14,23]. Hemodialysis, the primary treatment for 89% of end-stage renal disease patients, is associated with reduced health-related quality of life, high morbidity, and mortality, especially within the first year of treatment [2,3]. The economic burden of dialysis is substantial, and patients face frequent hospitalizations, heavy symptom burdens, and psychosocial challenges such as depression, anxiety, and social isolation [2]. Social support, a key modifiable factor, has been shown to improve outcomes and reduce mortality risk in this population [11,12,24].

Social support is a recognized factor influencing survival in patients with chronic kidney disease. Research highlights that social support, a modifiable psychosocial factor, can mitigate some of the negative outcomes associated with end-stage renal disease [11]. Dialysis patients often face psychosocial challenges, including depression, anxiety, and social isolation, which further reduce health-related quality of life [8,12]. McClellan et al. also identified social support as an independent predictor of mortality in dialysis patients, reinforcing its importance in this population [24]. Additionally, sleep disturbances, prevalent in patients with end-stage renal disease, are associated with depressive symptoms, increasing their risk of morbidity and mortality [12].

Our study revealed no significant differences between hemodialysis patients and healthy controls in the total MSSS score or its subscales, which assess tangible, emotional, informational, and affectionate support, as well as social interactions (Table 1). Similarly, the WHOQOL-BREF scores for Social Relationships and Environment domains did not differ significantly between the two groups (Table 1). These findings are notable, as previous studies suggest that dialysis patients often perceive lower social support due to the limitations imposed by their treatment [2,11,24]. However, our control group, composed of dialysis center employees and their families, represents a unique comparison cohort. These individuals, like dialysis patients, spend considerable time at the center or are separated from their loved ones during dialysis sessions. Thus, the reduced social support observed in dialysis patients may be related more to their absence from home and loved ones during treatment rather than solely their underlying kidney disease.

Notably, hemodialysis patients scored significantly lower in the Physical and Psychological domains of the WHOQOL-BREF compared to controls, indicating a poorer perception of their physical and mental health. The physical limitations imposed by end-stage renal disease, combined with the psychological strain of regular dialysis sessions, likely contribute to these findings [2,3]. The repetitive and prolonged absences from home required for hemodialysis sessions may exacerbate feelings of isolation and depression, further compromising health-related quality of life [3,8].

Gender differences in the perception of social support and quality of life were evident in our study. Males on hemodialysis scored lower in the Physical, Psychological, and Environmental domains of the WHOQOL-BREF, as well as in the MSSS subscales for affectionate support and positive social interactions compared to females on hemodialysis and their respective control groups (Table 2). In contrast, hemodialysis females reported higher scores in the MSSS subscales for tangible, emotional/informational, and affectionate support, suggesting that they perceive greater social support than their male counterparts (Table 2). This discrepancy may be due to the unique composition of our control group, where many females were dialysis center employees who shared similar environments with the patients, while the male controls were often alone at home. Thus, the quality of life in females may be less affected by dialysis-related absences, whereas males may experience greater psychological and environmental strain.

Interestingly, age did not significantly influence the assessment of social support or quality of life in our cohort (Table 2). This may reflect the small sample size of 115 patients, although it suggests that the impact of social support and health-related quality of life is relatively consistent across different age groups in the hemodialysis population.

Education appeared to affect only the Physical and Environmental domains of the WHOQOL-BREF, with higher scores observed among university graduates (Table 2). This could be attributed to the greater income levels in this group, providing better opportunities to manage physical health and improve environmental conditions. However, education did not influence social support scores in the MSSS, nor did it impact psychological health or social relationships, indicating that socioeconomic factors may play a limited role in perceived social support among dialysis patients. This is consistent with the high unemployment rates in this population, particularly in the United States, where over 75% of patients are unemployed at dialysis initiation [2].

Overall, our findings highlight the critical role of social support in managing the psychosocial and physical challenges of dialysis. Social support from family, friends, and healthcare professionals has been shown to reduce depressive symptoms and improve overall well-being [11,25]. Therefore, efforts to strengthen social support networks, particularly for dialysis patients who are single or lack close familial ties, are essential for enhancing health-related quality of life and improving health outcomes. Future guidelines for chronic kidney disease management should incorporate health-related quality of life and social support as key components of patient care [5].

Diabetes is a disease that extensively affects patients’ health, with its impact on the kidneys being just one of its many consequences [26]. In our group of dialysis patients, we found a statistically significant negative impact of diabetes on the Social Relationships and Environment domains of the WHOQOL-BREF test (Table 3). These findings align with broader research indicating that diabetic patients, particularly those undergoing dialysis, often experience a reduced quality of life due to the social and psychological burdens associated with managing this disease [27]. Studies further highlight that diabetes not only affects physical health but also increases social isolation and diminishes quality of life, particularly in areas of social interaction and environmental well-being [26]. Furthermore, the higher scores observed in the MSSS, MSSS-5, and Emotional/Informational and Positive Social Interactions subscales in patients with hypertension suggest that cardiologists may play a crucial role in providing these patients with the support they need. Research demonstrates that patients with hypertension often benefit from more frequent healthcare interactions, which can improve their social and emotional support, thus enhancing their overall quality of life [28]. The role of healthcare providers, especially cardiologists, in offering comprehensive care that addresses both physical and emotional needs is critical for improving outcomes in this patient group [26]. These findings are supported by the literature, which shows the broad impact of diabetes on quality of life, particularly in the Social and Environmental domains, and the potential benefits of structured support for hypertensive patients [28,29]. By addressing both the physical and social determinants of health, healthcare providers can play a pivotal role in managing chronic diseases and improving patient outcomes in dialysis populations.

Patients with chronic renal disease undergoing dialysis face significant and ongoing challenges, such as changes in their economic status, social roles, activity levels, and self-image, as well as impacts on their daily routines and health status [16]. The role of social support becomes crucial as these patients adjust to the realities of their condition. A robust social network can provide essential assistance, particularly in meeting the evolving needs of individuals undergoing long-term dialysis [16]. Hemodialysis patients, in particular, represent a vulnerable group with a strong need for social support from family, friends, and other caregivers [8]. The limitations imposed by lifelong dialysis often impair patients’ daily functioning, restricting social interactions and exacerbating their need for external support [8]. There is a well-established relationship between social support and resilience in chronic patients; social support enhances mental health and reduces stress [8]. Our findings further emphasize that longer dialysis sessions—especially those exceeding 4 h—are associated with lower scores in the WHOQOL-BREF Social Relationships domain, highlighting how prolonged treatment time affects patients’ social well-being (Table 4). While dialysis time did not influence other quality-of-life subscales, its impact on social relationships should prompt healthcare providers to consider the broader implications of prolonged dialysis on patients’ lives beyond just clinical outcomes.

Despite the technological advancements in treating chronic kidney disease, dialysis remains non-curative [14]. The perceived level of social support also appears to play a pivotal role in patients’ quality of life and treatment outcomes [14]. Accepting lifelong dialysis is challenging for patients, many of whom face limitations in daily activities and must adhere to a host of clinical and lifestyle requirements [4]. Several symptoms, such as fatigue, insomnia, and depression, further contribute to poor outcomes in dialysis patients [2]. In this context, social support has been consistently linked to improved health outcomes, not only in chronic kidney disease but also in other chronic diseases such as diabetes and heart disease [4]. Future research should focus on interventions aimed at strengthening social support systems, particularly from family members, significant others, and healthcare professionals, to improve treatment adherence and overall well-being in end-stage renal disease patients [4].

The impact of the type of hemodialysis access on social support and quality of life was not statistically significant in our study, except for an increase in the Tangible Support subscale for patients using central venous catheters (Table 4). This suggests that it is the dialysis treatment itself, rather than the type of access used, that influences social support and quality of life in patients. While arteriovenous fistulas and central venous catheters affect patient survival and complication rates, their role in social support appears minimal. Given the growing demand for patient-centered care, healthcare providers must prioritize innovation that improves not only longevity but also quality of life, with a focus on reducing symptom burden and enhancing social functioning [2]. High levels of social support have been associated with better physical and mental quality of life, fewer depressive symptoms, and lower rates of hospitalization [7]. However, our findings did not demonstrate a link between social support and either modality switching or mortality, suggesting that while social support plays a key role in enhancing patient experience and outcomes, it may not directly influence long-term survival [7].

The need for tailored social support interventions in dialysis care is underscored by the varying levels of support required by patients based on their social environment and disease severity [11]. Social support is an independent predictor of mortality in dialysis patients, with different aspects of support having variable importance depending on individual needs [11,12,24]. Clinical care providers should aim to incorporate programs such as self-help groups and psycho-educational interventions into patient care to enhance social support. Preparing patients psychologically for the demands of lifelong dialysis should also be a key component of clinical care [11]. Psychological resilience, bolstered by strong social support, has been shown to mitigate the depressive symptoms associated with dialysis and improve overall well-being [13]. Social networks, including family, friends, and healthcare professionals, play a vital role in promoting resilience and helping patients manage the stresses of chronic illness [13]. Importantly, patients with greater social support are more likely to adhere to treatment protocols, medications, and dietary restrictions, leading to better long-term outcomes [7]. Moreover, patients who identify as religious often report higher levels of perceived social support, likely due to their involvement in religious communities [15].

The MSSS-5 test demonstrates a high degree of correlation with the full MSSS test. The results suggest that even a shortened 5-item version can be a valuable tool for assessing social support in hemodialyzed patients, allowing for a quicker assessment when time is limited. Despite the limitations of using a shorter version, it can still provide meaningful insights into patients’ social support levels. Further studies may focus on confirming these findings in larger, multi-center trials, as well as investigating the test’s sensitivity to different patient subgroups or conditions. Ultimately, social support remains a key determinant of health-related quality of life in both hemodialysis and peritoneal dialysis patients, correlating with higher patient satisfaction, better-perceived health, and fewer hospitalizations [7]. However, the lack of association between social support and mortality or modality switching suggests that while it is a critical factor in improving patient-centered outcomes, its effects on long-term survival may be limited. More research is needed to explore the complex relationships between social support, patient outcomes, and healthcare costs in this population.

### Limitations

Several limitations of this study should be acknowledged. First, all data were based on patient self-reports, which may introduce subjective biases and potentially lead to a more one-sided assessment of social support and quality of life. Future research should consider using objective measures or collecting data from multiple family members to provide a more comprehensive understanding of social support dynamics. Second, this study was conducted in a single center, which limits the generalizability of the findings. The sample consisted only of adult patients undergoing chronic dialysis, leading to a relatively small sample size and reduced statistical power to detect subtle differences. Finally, the healthy control group predominantly comprised dialysis center employees and their relatives, individuals who frequently work long hours away from home, including nights, weekends, and holidays. This may have influenced the results and should be taken into consideration when interpreting the findings.

## 5. Conclusions

Hemodialysis patients, when compared to healthy individuals, reported lower scores in Physical and Psychological Health domains, though there were no significant differences in social support or cognitive function. The impact of social support and quality of life varies by gender. Male hemodialysis patients reported lower social support, physical health, psychological health, and environmental well-being, while female patients reported higher levels of social support but lower retrospective memory. Educational background also played a role, with university-educated hemodialysis patients reporting better physical health and environmental satisfaction. Additionally, married patients reported higher levels of social support.

Dialysis adequacy was found to influence certain cognitive functions, specifically planning and organization skills. Patients with arteriovenous fistulas as their vascular access point reported lower tangible support compared to those with other access types. The similar scoring of the full and abbreviated versions of the MSSS test supports the use of the 5-item MSSS-5 test when time is limited. However, the full version remains preferable whenever possible due to its ability to provide more detailed subscale data.

Incorporating the MSSS test into routine clinical practice could improve risk stratification among hemodialysis patients. The regular evaluation of social support and quality of life can facilitate early diagnosis and management of related symptoms. These findings enhance our understanding of the social and psychological challenges faced by hemodialysis patients, enabling healthcare providers to intervene earlier and mitigate the impact of these challenges on patient well-being.

## Figures and Tables

**Table 1:** Modified Social Support Survey (MSSS) and World Health Organization Quality of Life (WHOQOL-BREF) questionnaires.

			HD Patients	Healthy Controls	
		Scoring Range	n/M [Range] (SD)	n/M [Range] (SD)	*p*-Value
sex	male		79 (68.7%)	72 (67.3%)	N/S
	female		36 (31.3%)	35 (32.7%)	N/S
age			63.7 [31–86] (11.5)	56.2 [30–73] (10.8)	N/S
Modified Social Support Survey (MSSS)	MSSS total score	0–100	71.5 (26.2)	69.9 (26.3)	N/S
	5-item MSSS-5 score	0–100	72.4 (26.7)	70.8 (26.8)	N/S
	Tangible Support Subscale	0–100	77.3 (25.9)	72.9 (26.8)	N/S
	Emotional/Informational Support Subscale	0–100	69.2 (27.4)	66.3 (28.3)	N/S
	Affectionate Support Subscale	0–100	71.2 (30.7)	71.6 (27.8)	N/S
	Positive Social Interaction Subscale	0–100	68.3 (29.0)	69.0 (27.6)	N/S
World Health Organization Quality of Life questionnaire (WHOQOL-BREF)	Physical health domain	4–20	**↓** **12.0 (1.8)**	**↑ 13.1 (1.5)**	**<0.0001**
	Psychological health domain	4–20	**↓** **12.7 (2.1)**	**↑ 14.2 (1.8)**	**<0.0001**
	Social relationship domain	4–20	13.5 (3.1)	13.6 (3.3)	N/S
	Environment domain	4–20	13.7 (2.2)	14.1 (2.6)	N/S

HD—hemodialysis; N/S—*p* > 0.05.

**Table 2:** Demographic factors affecting MSSS and WHOQOL-BREF scores.

Questionnaires		Sex						Age			Level of Education				Marital Status		
		HD Male	Healthy Male	HD vs. Healthymalep-Value	HD Female	Healthy Female	HD vs. Healthy female *p*-Value	<Median	>Median	*p*-Value	Primary	Secondary	University	*p*-Value	Married	Single	*p*-Value
**MSSS**	MSSS total score	**↓** **68.8 (26.3)**	**↑ 80.0 (26.9)**	**<0.01**	**↑ 77.4 (25.5)**	**↓** **62.7 (23.8)**	**<0.02**	70.7 (29.4)	72.7 (22.9)	N/S	71.9 (25.7)	73.2 (26.7)	66.6 (28.1)	N/S	**↑ 75.7 (22.7)**	**↓** **62.9 (30.8)**	**<0.02**
	5-item MSSS-5 score	**↓** **68.9 (27.2)**	**↑ 80.8 (27.2)**	**<0.009**	**↑ 80.1 (24.2)**	**↓** **63.5 (24.4)**	**<0.006**	71.0 (30.9)	74.1 (22.0)	N/S	72.6 (26.1)	74.7 (27.3)	67.1 (28.5)	N/S	**↑ 76.5 (22.6)**	**↓** **64.1 (32.3)**	**<0.02**
	Tangible Support Subscale	74.3 (26.5)	77.2 (32.4)	N/S	**↑ 83.9 (23.5)**	**↓** **69.8 (21.8)**	**<0.02**	73.1 (30.7)	81.8 (19.4)	N/S	75.1 (26.1)	80.5 (27.3)	79.0 (22.7)	N/S	80.4 (22.9)	71.7 (30.5)	N/S
	Emotional/Informational Support Subscale	65.9 (28.3)	74.4 (32.3)	N/S	**↑ 76.4 (24.1)**	**↓** **60.5 (23.9)**	**<0.007**	67.2 (30.5)	71.5 (24.1)	N/S	69.1 (28.9)	71.5 (24.7)	64.7 (27.5)	N/S	**↑ 72.8 (24.8)**	**↓** **61.9 (31.0)**	**<0.05**
	Affectionate Support Subscale	**↓** **67.9 (30.0)**	**↑ 87.2 (21.7)**	**<0.0001**	**↑ 78.5 (31.4)**	**↓** **60.2 (26.5)**	**<0.01**	72.4 (33.3)	70.5 (28.2)	N/S	71.4 (31.1)	74.0 (28.4)	65.2 (34.3)	N/S	**↑ 76.0 (27.8)**	**↓** **61.6 (34.3)**	**<0.02**
	Positive Social Interaction Subscale	**↓** **67.0 (29.5)**	**↑ 81.1 (24.8)**	**<0.002**	71.1 (28.1)	60.2 (26.6)	N/S	69.9 (31.3)	67.0 (26.9)	N/S	72.0 (27.1)	66.7 (30.7)	57.4 (31.3)	N/S	**↑ 73.8 (25.7)**	**↓** **57.0 (32.3)**	**<0.005**
**WHOQOL-BREF**	Physical health Domain	**↓** **12.1 (1.6)**	**↑ 13.7 (1.2)**	**<0.0001**	11.8 (2.1)	12.7 (1.6)	N/S	12.1 (1.7)	12.0 (1.9)	N/S	**↓** **11.9 (1.8)**	**↓** **11.8 (1.8)**	**↑ 13.0 (1.5)**	**<0.04 (prim. vs. univ.)** **<0.03 (second. vs. univ.)**	12.1 (1.9)	11.9 (1.4)	N/S
	Psychological health Domain	**↓** **12.9 (2.2)**	**↑ 14.7 (1.9)**	**<0.0001**	12.3 (2.0)	13.9 (1.7)	N/S	12.7 (2.1)	12.8 (2.1)	N/S	12.6 (2.2)	12.8 (2.1)	13.2 (1.9)	N/S	13.0 (2.1)	12.3 (2.1)	N/S
	Social Relationship Domain	13.4 (3.1)	13.4 (3.4)	N/S	13.9 (3.1)	13.7 (3.2)	N/S	13.3 (3.4)	13.8 (2.8)	N/S	13.3 (3.4)	13.8 (2.8)	14.0 (2.4)	N/S	13.8 (3.0)	13.1 (3.4)	N/S
	Environment Domain	**↓** **13.8 (2.2)**	**↑ 15.2 (3.1)**	**<0.002**	13.5 (2.2)	13.3 (2.0)	N/S	13.6 (2.4)	13.8 (2.1)	N/S	**↓** **13.4 (2.3)**	**↓** **13.6 (2.0)**	**↑ 15.1 (1.8)**	**<0.006 (prim. vs. univ.)** **<0.01 (second. vs. univ.)**	13.8 (2.2)	13.4 (2.2)	N/S

MSSS—Modified Social Support Survey; MSSS-5—5-item MSSS version; WHOQOL-BREF—World Health Organization Quality of Life questionnaire. N/S—*p* > 0.05.

**Table 3:** Comorbidities affecting MSSS and WHOQOL-BREF scores.

Questionnaires		Diabetes			Hypertension		
		Yes	No	*p*-Value	Yes	No	*p*-Value
**MSSS**	MSSS total score	65.5 (36.6)	72.5 (33.1)	N/S	**↑** **79.7 (28.5)**	**↓** **57.4 (34.0)**	**<0.01**
	5-item MSSS-5 score	55.7 (36.3)	68.9 (34.0)	N/S	**↑** **72.6 (31.8)**	**↓** **55.5 (33.2)**	**<0.01**
	Tangible Support Subscale	55.7 (35.6)	64.9 (34.5)	N/S	**↑** **70.9 (31.6)**	**↓** **49.2 (33.0)**	**<0.02**
	Emotional/Informational Support Subscale	52.0 (37.9)	66.7 (38.1)	N/S	68.1 (35.8)	56.1 (39.0)	N/S
	Affectionate Support Subscale	50.4 (38.8)	63.3 (36.5)	N/S	67.2 (36.0)	49.6 (32.8)	N/S
	Positive Social Interaction Subscale	55.9 (36.0)	66.8 (33.2)	N/S	**↑** **71.5 (31.4)**	**↓** **53.1 (31.3)**	**<0.03**
**WHOQOL-BREF**	Physical health Domain	11.5 (1.9)	12.0 (1.7)	N/S	12.0 (1.8)	11.7 (1.6)	N/S
	Psychological health Domain	11.9 (2.1)	12.7 (2.5)	N/S	12.7 (2.2)	11.9 (2.7)	N/S
	Social Relationship Domain	**↓** **11.9 (3.5)**	**↑** **14.1 (2.8)**	**<0.01**	13.8 (2.9)	12.7 (3.6)	N/S
	Environment Domain	**↓** **12.6 (2.4)**	**↑** **13.9 (2.3)**	**<0.04**	13.6 (2.4)	13.5 (2.4)	N/S

MSSS—Modified Social Support Survey; MSSS-5—5-item MSSS version; WHOQOL-BREF—World Health Organization Quality of Life questionnaire. N/S *p* > 0.05.

**Table 4:** Dialysis-related factors affecting MSSS and WHOQOL-BREF scores.

Questionnaires		Duration of HD Session			Months on HD		Kt/V			Vascular Access			URR		
		<4h	>4h	*p*-Value	Correlation	*p*-Value	<Median	>Median	*p*-Value	AVF	CVC	*p*-Value	<Median	>Median	*p*-Value
**MSSS**	MSSS total score	75.3 (26.5)	69.2 (21.4)	N/S	0.18	N/S	73.3 (25.0)	69.2 (27.5)	N/S	70.5 (26.8)	79.1 (20.1)	N/S	73.4 (24.3)	70.7 (27.2)	N/S
	5-item MSSS-5 score	78.3 (24.9)	68.2 (22.4)	N/S	0.18	N/S	73.3 (26.0)	71.0 (27.5)	N/S	71.1 (27.6)	82.3 (16.2)	N/S	73.6 (25.8)	72.3 (26.8)	N/S
	Tangible Support Subscale	83.1 (23.4)	70.5 (28.9)	N/S	0.16	N/S	78.6 (26.9)	77.4 (25.1)	N/S	**↓** **75.4 (26.5)**	**↑** **92.3 (12.5)**	**<0.02**	76.9 (25.3)	78.8 (25.4)	N/S
	Emotional/Informational Support Subscale	75.3 (25.7)	64.5 (27.4)	N/S	0.18	N/S	70.3 (28.4)	67.5 (26.4)	N/S	68.3 (28.0)	76.2 (21.3)	N/S	70.7 (27.7)	68.8 (26.2)	N/S
	Affectionate Support Subscale	74.2 (33.8)	71.6 (30.0)	N/S	0.20	N/S	73.2 (28.9)	68.7 (32.5)	N/S	70.3 (31.5)	78.8 (23.2)	N/S	73.2 (29.0)	70.3 (31.6)	N/S
	Positive Social Interaction Subscale	68.4 (30.0)	70.5 (20.4)	N/S	0.19	N/S	72.8 (26.0)	63.2 (31.1)	N/S	68.1 (29.4)	69.2 (26.4)	N/S	72.7 (25.5)	64.9 (31.1)	N/S
**WHOQOL-BREF**	Physical health Domain	12.4 (1.9)	11.9 (1.7)	N/S	0.10	N/S	12.2 (1.6)	11.9 (2.0)	N/S	12.0 (1.8)	12.5 (1.7)	N/S	12.0 (1.6)	12.1 (2.0)	N/S
	Psychological health Domain	13.1 (2.4)	12.8 (1.9)	N/S	0.10	N/S	13.2 (1.9)	12.3 (2.3)	N/S	12.8 (2.2)	12.4 (1.9)	N/S	13.0 (2.1)	12.5 (2.1)	N/S
	Social Relationship Domain	**↑** **14.7 (3.4)**	**↓** **12.8 (2.7)**	**<0.02**	0.10	N/S	13.9 (2.7)	13.2 (3.5)	N/S	13.6 (3.0)	13.3 (3.7)	N/S	13.6 (3.1)	13.5 (3.1)	N/S
	Environment Domain	14.1 (2.0)	14.0 (1.9)	N/S	0.18	N/S	14.1 (2.0)	13.3 (2.4)	N/S	13.7 (2.2)	13.5 (2.5)	N/S	13.8 (2.1)	13.7 (2.3)	N/S

HD—hemodialysis; AVF—arteriovenous fistula; CVC—central venous catheter; URR—urea reduction ratio; MSSS—Modified Social Support Survey; MSSS-5—5-item MSSS version; WHOQOL-BREF—World Health Organization Quality of Life questionnaire. N/S *p* > 0.05.

## Data Availability

The data presented in this study are available on request from the corresponding author. The data are not publicly available due to privacy restrictions.
